# Effects of Anode Flow Field Design on CO_2_ Bubble Behavior in μDMFC

**DOI:** 10.3390/s90503314

**Published:** 2009-05-06

**Authors:** Miaomiao Li, Junsheng Liang, Chong Liu, Gongquan Sun, Gang Zhao

**Affiliations:** 1 Key Laboratory for Micro/Nano Technology and System of Liaoning Province, Dalian University of Technology, Dalian, Liaoning Province, 116023, P.R. China; E-Mail: limiaomiao_mems@163.com (M.L.); 2 Key Laboratory for Precision and Non-traditional Machining Technology of Ministry of Education, Dalian University of Technology, Dalian, Liaoning Province, P.R. China; 3 Direct Alcohol Fuel Cell Laboratory, Dalian Institute of Chemical Physics, Chinese Academy of Science, Dalian, Liaoning Province, P. R. China

**Keywords:** μDMFC, flow field, pressure drop, CO_2_ bubbles

## Abstract

Clogging of anode flow channels by CO_2_ bubbles is a vital problem for further performance improvements of the micro direct methanol fuel cell (μDMFC). In this paper, a new type anode structure using the concept of the *non-equipotent serpentine flow field* (*NESFF*) to solve this problem was designed, fabricated and tested. Experiments comparing the μDMFC with and without this type of anode flow field were implemented using a home-made test loop. Results show that the mean-value, amplitude and frequency of the inlet-to-outlet pressure drops in the *NESFF* is far lower than that in the traditional flow fields at high μDMFC output current. Furthermore, the sequential images of the CO_2_ bubbles as well as the μDMFC performance with different anode flow field pattern were also investigated, and the conclusions are in accordance with those derived from the pressure drop experiments. Results of this study indicate that the *non-equipotent* design of the μDMFC anode flow field can effectively mitigate the CO_2_ clogging in the flow channels, and hence lead to a significant promotion of the μDMFC performance.

## 1. Introduction

Recently, the micro direct methanol fuel cell (μDMFC) fabricated using microtechnologies [1] has drawn increasing interest for power applications in advanced portable electronics because of its benefits such as low pollution emission, high theoretical power density [2], high efficiency [3] and relatively simple system features [4-7]. In DMFC, the anode flow field works as the fuel distributor, which delivers methanol solution uniformly along the electrode surface. Furthermore, micro channels in the anode flow field also provide suitable sites for mass transportation and exchange between reactants and byproducts, such as methanol solution and carbon dioxide. During the electrochemical reaction of the DMFC, the carbon dioxide is produced as byproduct from the gas diffusion layer of the membrane-electrode-assembly (MEA), then mixes with aqueous methanol solution and outflows from the fuel cell via anode channels. CO_2_ bubbles in the anode flow field may block the transportation of methanol and occupy the effective anodic catalytic oxidation sites, finally deteriorating the DMFC's performance [8-12]. Therefore, the structure of anode flow field need to be optimized in order to mitigate the clogging of CO_2_ bubbles and thus improve fuel cell performance.

Work on CO_2_ bubble behavior has attracted researchers' attention in recent years. Simulated CO_2_ behavior based on the decomposition of hydrogen peroxide solution (H_2_O_2_) in the anode flow field was studied by Bewer [13], who found that the flow field with grid structures gave a better bubble transport effect in large-size DMFC. Yang *et al.* [14,15] carried out a visual study on the CO_2_ bubble behavior under different current density and methanol flow rates using an in-house fabricated transparent DMFC. After experimentally investigating the pressure drop of the two-phase flow in the anode flow fields, they claimed that the pressure drop increased with higher current density at the low value scope, but after the current density reached a peak, the trends of pressure drop was reversed. Liao *et al.* [16] also reported a visualization study on the dynamics of CO_2_ bubbles in anode channels and the performance of a DMFC. It was observed in their study that the processes of emergence, growth, coalescence, detachment, and sweeping of the gas bubbles always occurred periodically. Besides experimental studies, many model-based mathematic simulations on the two-phase flow characteristics of the DMFC anode flow field were also proposed in the literature. For example, Kulikovsky [17] has recently built a 1D+1D model of DMFC, which taken into account gaseous bubbles in the anode channel. By deriving the asymptotic solution to the model equations for the case of small rate of bubbles formation, the author obtained the formula for the change in the mean current density of the cell due to the behavior variation of the CO_2_ bubbles. Maharudrayya *et al.* [18] investigated the flow distribution and pressure drop in the DMFC anode flow field using a combination of CFD simulation and experiments. They concluded that multiple Z-type configurations had a lower non-uniformity in flow index and higher pressure drop. The results from the mathematic model provide parameterized characterization of the bubbles behavior and their influences on the DMFC performance.

The above mentioned research has provided effective methods to investigate the behavior of CO_2_ bubbles and mitigate their adverse impact on performance of the DMFC. However, with the significant feature sizes decreasing of the anode flow channels in μDMFC, especially the hydraulic diameter of micro channels is less than 1 mm, CO_2_ clogging may become a vital limitation for a further performance improvements of the fuel cell [19]. In order to mitigate the clogging of the CO_2_ bubbles in the middle and export region of the flow field [14], a new anode flow field pattern for μDMFC with gradual change in width along the micro channel was demonstrated in the present work. We call this type of flow field as *non-equipotent flow field* in order to distinguish it from the conventional flow field patterns. Transparent μDMFC single cells with and without this flow field pattern were fabricated and comparatively studied. Preliminary results showed that the μDMFC performance was effectively promoted using the designing concept of *non-equipotent flow field*.

## 2. Experimental details

### 2.1. Structure of the Transparent μDMFC Single Cell

**Figure 1.**
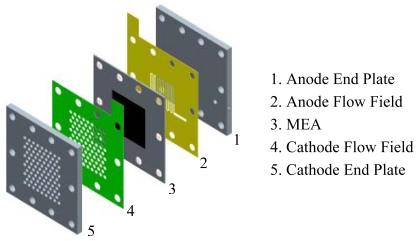
Infrastructure of μDMFC.

In this work, transparent μDMFCs were designed and fabricated. The fuel cells ran on aqueous methanol solution which was driven by a syringe pump in the anode and absorbed oxygen from the ambient air (air-breathing mechanism) in the cathode. As shown in Figure.1, the μDMFC consists of MEA, flow field plates and end plates. The MEA with an active area of 1.4 cm × 1.4 cm was sandwiched between two flow field plates, which were sealed with PTFE gaskets on both sides. The MEA was supplied by Dalian Institute of Chemical Physics, the fabrication process was described in our previous work [20]. The flow field plates were made of stainless steel sheet (SS316L, 400 μm in thickness) using double-sides photochemical micro fabrication techniques. The end plates were made from 2mm thick polymethyl methacrylate (PMMA) sheets using a laser milling method.

### 2.2. Flow Field Design and Fabrication

**Figure 2.**
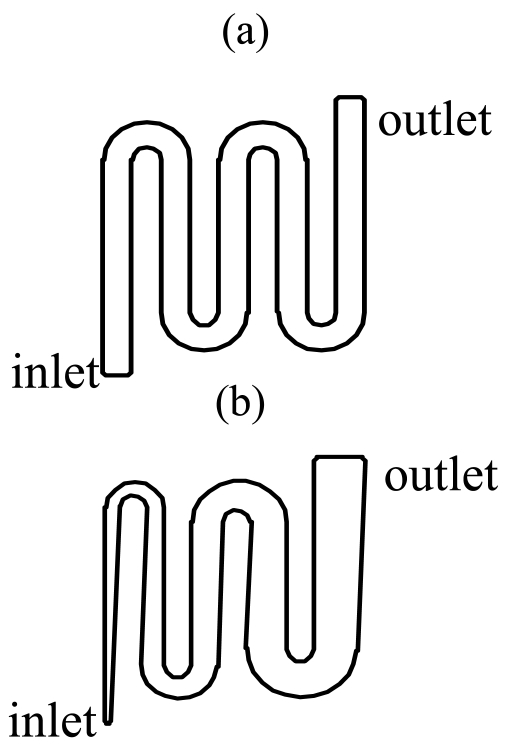
ESFF (a) and NESFF (b) patterns.

**Figure 3.**
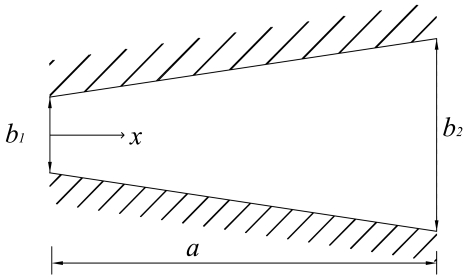
Geometric relations of NESFF channel.

Two types of anode flow field patterns were designed in this study (see Figure 2), one is the *equipotent serpentine flow field* (*ESFF*), the other is the *non-equipotent serpentine flow field* (*NESFF*). Traditional dot matrix flow field pattern was adopted on the cathode side. The detailed geometry of each anode flow field is shown in Table 1. As can be seen from Figure 2 and Table 1, the *ESFF* and *NESFF* consisted of a single meandering flow channel, each of which has a total length of 70.0 mm. The channel width of the *NESFF* gradually changed along the channel length. In the present work, the effects of the *ESFF* and *NESFF* patterns on the CO_2_ bubbles behavior were compared on the basis of the same hydraulic diameter and total length of the channels, as well as the same open ration and rib width of the flow fields. Herein, the open ratio is defined as the ratio of the flow channel area to the total MEA area (1.96 cm^2^), and the hydraulic diameter of the taper channel in *NESFF* is an equivalent value, which can be calculated from the geometric relations shown in Figure 3.

In Figure.3, the *NESFF* channel width gradually changing along the channel length *x* can be calculated as:

(1) $$b(x)=\frac{b_{2}-b_{1}}{a}x+b_{1}$$

Defining the average hydraulic diameter along *x* as the equipotent hydraulic diameter of the *NESFF* channel, then we get:

(2) $$Dx=\frac{1}{a}\int_{0}^{a}D_{x}(x)\text{dx}=\frac{1}{a}\int_{0}^{a}\frac{4A(x)}{P(x)}\text{dx}=\frac{1}{a}\int_{0}^{a}\frac{4c\times b(x)}{2[c+b(x)]}\text{dx}$$

where *D_x_* is the equipotent hydraulic diameter, *D_x_*(*x*) is hydraulic diameter per unit length, *a* is channel length, *c* is channel depth, *b*(*x*) is channel width, *b*_2_ and *b*_1_ are the outlet and inlet width of channel, respectively.

**Figure 4.**
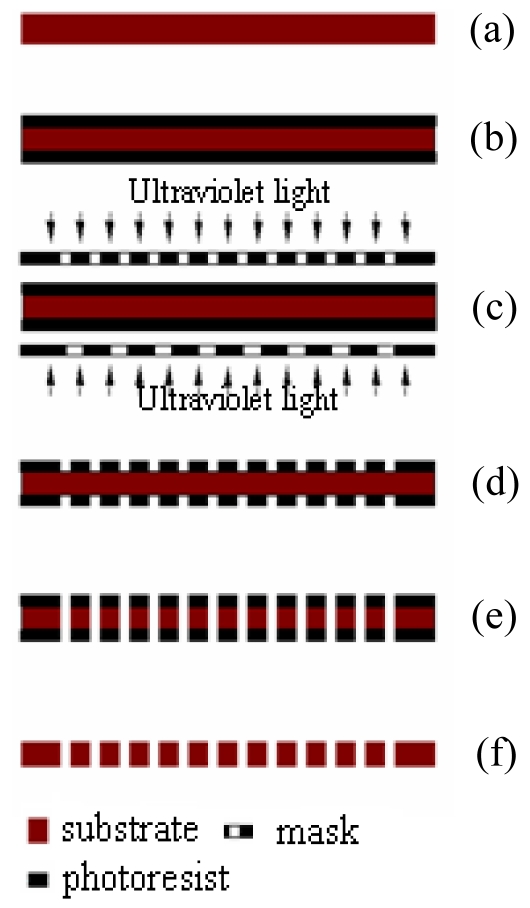
Etching process of the flow field plate.

The flow field plates with a minimum channel dimension of 400 μm were fabricated using double-sided micro photochemical etching. The fabrication sequence is shown in Figure 4. The cleaned and dried stainless steel substrate (SS316L) (a) was spin-coated with one SU-8 photoresist layer with a thickness of 10 μm on both sides (b). Then, the flow field patterns on the mask were transferred to the photoresist layers using UV-based lithography techniques (c), and developed using a 1% sodium carbonate developer (d). The flow field patterns were obtained after etching in FeCl_3_-HCl for 30 min (e), and the residual photoresist was removed using NaOH solution at 50°C (f).

### 2.3. Set-up of the Test Loop

**Figure 5.**
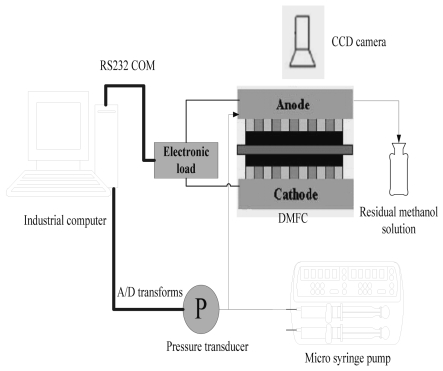
Schematic of the μDMFC test loop.

In this study, the effects of CO_2_ bubbles were characterized using the inlet-to-outlet pressure drop and the two-phase flow behavior in the channels of anode flow field. On the other hand, the performance of the μDMFC was examined using an electronic load. Figure 5 shows the schematic drawing of the experimental test loop. In the test loop, a high accuracy syringe pump (Medical Instrument Corporation of Zhejiang University, WZS-50F2) with double channels was used to deliver methanol solution to the μDMFC. A programmable electronic load (Itech Electronics, IT8511) was adopted to simulate the variation of the external load during the test. The inlet-to-outlet pressure drop in anode flow field was monitored with a pressure transmitter (Beijing Westzh M&E Technology Ltd, CYB-3051). The CCD camera (Toshiba Teli Corporation, CSFU15BC18) was adopted to record the two-phase flow behavior in the flow channels. The experimental data and flow behavior were acquired and analysed by a real-time computer.

It should be noted that all the experiments in this work were conducted under the following conditions: (1) The μDMFCs were horizontally located on the test position with the anode side upward, (2) The concentration of the methanol solution was 3 mol/L, (3) The methanol solution was fed into the anode flow field with a constant flow rate of 3 mL/h at an ambient temperature of 25°C.

## 3. Results and discussion

### 3.1. Anode Pressure Drop of the μDMFC

**Figure 6.**
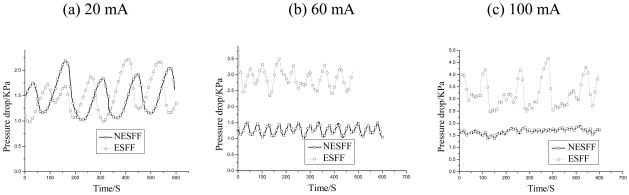
Pressure drops of ESFF and NESFF at different current.

Figure 6 shows the time-history curves of the inlet-to-outlet pressure drops in the SEFF and NSEFF. The pressure drops were measured at currents of 20, 60 and 100 mA, respectively. It was observed that the pressure drops curves of the ESFF and NESFF were both in a periodically oscillatory form during the electrochemical reaction process. However, the pressure drop histories in NESFF and ESFF showed very different features with the change of the fuel cell current. It can be found from Figure.6(a) that the mean-value, amplitude and frequency of the pressure drop curves of NESFF and ESFF were very close at the current of 20 mA, because that the flow patterns in anode flow field were mainly bubbly flow at low fuel cell current, which conceal the bubble clogging effects in the micro channels. But when the current increased, the different trends of the pressure drop in NESFF and ESFF appeared [Figures 6(b) and 6(c)].

**Figure 7.**
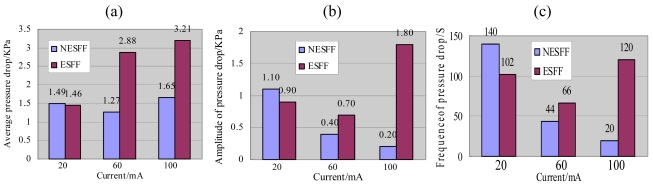
The histogram of the statistical pressure drops: (a) the average pressure drop, (b) the amplitude of pressure drop, and (c) the frequencies of pressure drop.

Figure 7 is the corresponding histogram of the statistical pressure drops results obtained from Figure 6. It was also indicated that the pressure drops in the anode flow field were strongly dependent on the output current of the μDMFC. When the fuel cell current was adjusted from 20 mA to 100 mA, the average pressure drops in NESFF and ESFF about 1.49 KPa and 1.46 KPa to 1.65 KPa and 3.2KPa, were obtained, respectively [Figure 7(a)]. This can be explained by the fixed stoichiometry relationship between the mole number of the electron and CO_2_ in the μDMFC electrochemical reactions. It is clear from Figure.7(a) that the average pressure drop in NESFF only had a 10.7% increase (from 1.49KPa to 1.65KPa) when the fuel cell current varied from 20mA to 100mA. Meanwhile, the pressure drop in ESFF increased more than 2 folds from 1.46KPa to 3.21KPa under the same circumstances. On the other hand, the amplitude and frequency of the pressure drop curves (Figure.7 (b) and Figure.7 (c)), which were reflecting the periodical accumulation and elimination of the CO_2_ bubbles in the micro channels, also had quite different tendencies with the change of fuel cell current. It is clear from Figure.7 (b), when the fuel cell current was increased from 20mA to 60mA and 100mA, the amplitude of the NESFF pressure drop decreased from 1.1KPa to 0.4KPa and 0.2KPa. However, the amplitude of the ESFF pressure drop decreased first from 0.9KPa to 0.7KPa, and then increased afterwards to 1.8KPa. It means that in the NESFF, the more CO_2_ is released to the channel, the less fluctuation of the methanol transportation is realized. But for the ESFF, the accumulation of CO_2_ may lead to a serious block of the anode channel and unstable methanol transportation. This conclusion also is proved by the pressure drop frequency vs. the fuel cell current that shown in Figure.7(c), which has similar trend comparing with that of the pressure drop amplitude. The above mentioned pressure drop differences imply that the non-equipotent design of the μDMFC anode flow field can effectively mitigate the CO_2_ clogging in the anode flow channels.

### 3.2. CO_2_ Bubble Behavior Analysis

**Figure 8.**
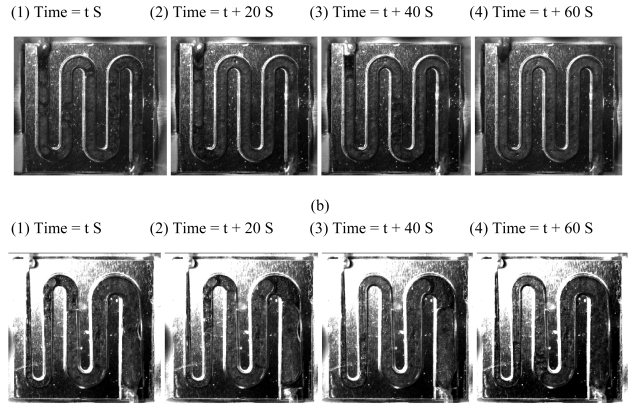
CO_2_ gas bubble behavior at the current of 100mA in the ESFF (a) and NESFF (b).

In order to obtain a better understanding of the features of the CO_2_ bubbles in the μDMFC, an image of the anode flow field was acquired using a real-time image acquisition system. The conditions of this experiment were similar to that of the pressure drop experiments. Before the image acquisition process, the output current of the μDMFC was firstly adjusted to a fixed value for 5 minutes until the fuel cell voltage was stable. The anode flow field images were sequentially recorded at a rate of 15 frames/s. Considering to the length limitation, only the sequential images at 100 mA with a time interval of 20 s are shown in this paper. Figures 8(a) and 8(b) are the anode images of the μDMFC with ESFF and NESFF patterns in a cycle of the pressure drop vibration, respectively. It can be found from Figure 8 that the shapes and distribution of the CO_2_ bubbles in the ESFF were more anomalous compared with those of the CO_2_ bubbles in the NESFF. This is because the tapered channels in the NESFF can provide additional confines to the coalescence and movement of the CO_2_ bubbles, which may in turn lead to a more orderly bubble behavior in the anode flow field. This result is in accordance with that of the pressure drop experiment mentioned above. On the other hand, the effects of different anode flow field pattern on the CO_2_ bubble accumulation and elimination also can be evaluated with the residual volume fraction (RVF) of bubbles in the anode flow field. Table 2 compares the RVF of bubbles in ESFF and NESFF at 20 mA, 60 mA and 100 mA. Results show that the RVF of bubbles in the two anode flow fields was similar at low fuel cell current (20 mA, 60 mA), but when the current increased to 100 mA, the RVF of bubbles in ESFF became strongly fluctuating (from 60%∼100%). Meanwhile, the RVF of bubbles in NESFF kept stable (approximately from 74% to 84%) even at a fuel cell current of 100mA. These experimental results also confirm the benefits of the NESFF to remove the CO_2_ bubbles in the anode flow field of the μDMFC.

### 3.3. Performance Evaluation

**Figure 9.**
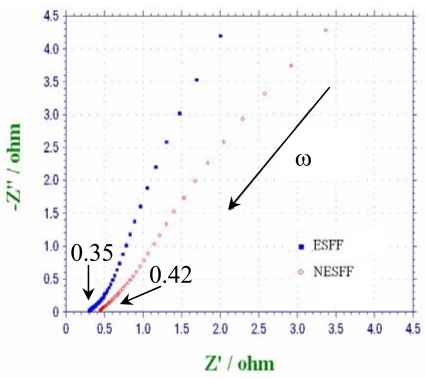
Nyquist plot of μDMFCs impedance with NESFF and ESFF

**Figure 10.**
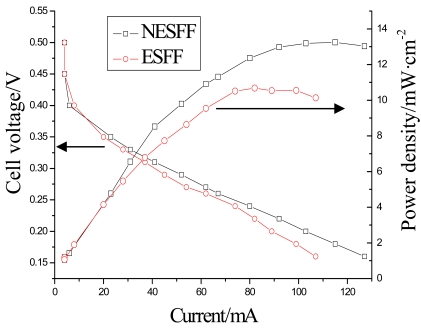
Performance of the μDMFCs with NESFF and ESFF

In order to further prove the benefits of the NESFF, μDMFC performance with and without this kind of flow field pattern were compared in this study. The fuel cell performance usually can be evaluated using a polarization curve (IV curve). However, the difference of the internal resistances between these two fuel cells should be adjusted to an acceptable scope before the performance evaluation. Figure 9 shows the Nyquist plot of the μDMFC impedance with and without NESFF in anode, which was obtained by using an electrochemical workstation (CHI660A, CHInstruments Inc.). Results show that the internal ohmic resistance of the μDMFC with ESFF and NESFF patterned anodes were 350 mΩ and 420 mΩ, respectively. The slight difference of the internal resistance is due to different assembly conditions of the fuel cells. Figure 10 presents the performance of the μDMFCs equipped with NESFF and ESFF using 3 M methanol solution at a flow rate of 3mL/h (temperature is 25°C). Moreover, as it can be seen from Figure 10, the power density difference between the two μDMFCs was enlarged with the increase of the fuel cell current (also means the increase of the quantity of the CO_2_), and the peak absolute power of the NESFF μDMFC (25.97 mW) was 24% higher than that of the ESFF μDMFC (20.91 mW). This may also be attributed to the effects of the anode flow field pattern on the CO_2_ clogging behavior that have been analyzed in above paragraphs.

## 4. Conclusions

This paper reports on the effects of anode flow field design on CO_2_ bubble clogging. A new NESFF pattern of the anode flow field with a gradual change in width along the micro flow channel was designed. Transparent μDMFC fuel cells equipped with NESFF and ESFF were fabricated and compared. Experimental results showed that the pressure drops of the ESFF and NESFF were both in a periodically oscillatory form during the electrochemical reaction process, but the pressure drops in NESFF and ESFF showed very different responses to fuel cell current changes, which implied that *non-equipotent flow field* can obtain a more uniform distribution of the CO_2_ bubbles. The benefits of the NESFF to remove the CO_2_ bubbles were also confirmed by CO_2_ bubble behavior analysis. It can be found that the peak absolute power of the NESFF μDMFC is 24% higher than that of the ESFF μDMFC. The power density difference between the two μDMFC was enlarged with the increase of the fuel cell current. It can be concluded that the *non-equipotent flow field* design was beneficial for the removal of CO_2_ bubbles, and thus led to a higher performance of the μDMFC.

## Figures and Tables

**Table 1.** Geometry of the flow fields.

**Flow fields**	**Channel depth(mm)**	**Channel Inlet Width(mm)**	**Channel Outlet Width(mm)**	**Channel Taper**	**Channel length(mm)**	**Open ratio(%)**	**Hydraulic diameter(mm)**
ESFF	0.4	1.56	1.56	1:1	70	56	0.58
NESFF	0.4	0.4	2.9	1:28.6	70	58	0.58

**Table 2.** The residual volume fraction of bubbles.

**Current**	**ESFF (t s)**	**ESFF (t+20 s)**	**ESFF (t+60 s)**	**ESFF (t+100 s)**	**NESFF (t s)**	**NESFF (t+20 s)**	**NESFF (t+60 s)**	**NESFF (t+100 s)**
20 mA	42.33%	47.50%	35.97%	20.46%	20.56%	22.63%	21.48%	22.86%
60 mA	40.30%	58.04%	51.97%	47.96%	50.44%	52.93%	34.71%	59.60%
100 mA	60.47%	90.27%	82.25%	100%	83.76%	73.66%	80.03%	83.42%
